# QuickStats

**Published:** 2013-04-12

**Authors:** Debra Blackwell, Tainya C. Clarke

**Figure:**
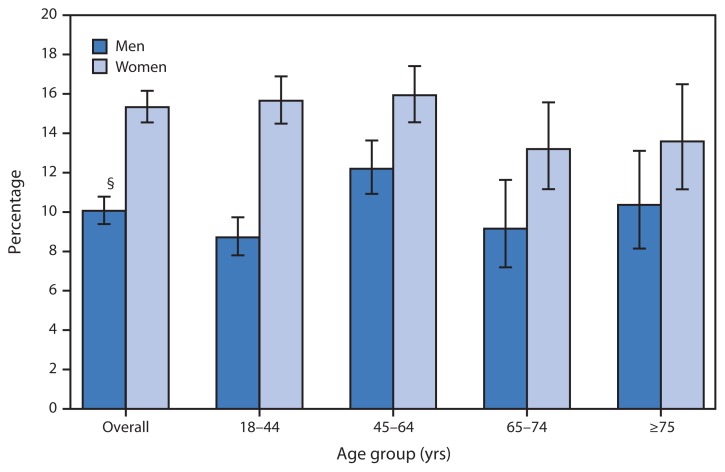
Percentage of Adults Who Often Felt Very Tired or Exhausted in the Past 3 Months,* by Sex and Age Group — National Health Interview Survey, United States, 2010–2011^†^ * Based on responses to the following: “In the past 3 months, how often did you feel very tired or exhausted? Would you say never, some days, most days, or every day?” Persons reporting feelings of tiredness or exhaustion on most days or every day were categorized as often feeling very tired or exhausted. Unknowns were not included in the denominators when calculating percentages. ^†^ Estimates are based on household interviews of a sample of the U.S. civilian, noninstitutionalized population. ^§^ 95% confidence interval.

During 2010–2011, women (15.3%) were more likely than men (10.1%) to often feel very tired or exhausted. Among adults aged 18–44 years, women were nearly twice as likely as men (15.7% versus 8.7%) to often feel very tired or exhausted. In addition, a difference was observed among women and men aged 45–64 years (15.9% versus 12.2%), but no differences by sex were observed among persons aged 64–74 years or those aged ≥75 years.

**Source:** National Health Interview Survey, 2010 Quality of Life and 2011 Functioning and Disability supplements. Data were from a subset of the adults randomly selected for the Sample Adult Component of the National Health Interview Survey questionnaire. Additional information available at http://www.cdc.gov/nchs/nhis.htm.

